# The cell adhesion molecule Fasciclin2 regulates brush border length and organization in *Drosophila* renal tubules

**DOI:** 10.1038/ncomms11266

**Published:** 2016-04-13

**Authors:** Kenneth A. Halberg, Stephanie M. Rainey, Iben R. Veland, Helen Neuert, Anthony J. Dornan, Christian Klämbt, Shireen-Anne Davies, Julian A. T. Dow

**Affiliations:** 1 Institute of Molecular, Cell and Systems Biology, College of Medical, Veterinary and Life Sciences, University of Glasgow, Davidson Building Room 324, Glasgow G12 8QQ, UK; 2 Section for Cell & Neurobiology, Department of Biology, University of Copenhagen, Universitetsparken 15, Copenhagen DK-2100, Denmark; 3 MRC—University of Glasgow Centre for Virus Research, Henry Wellcome Building, 464 Bearsden Road, Glasgow G61 1QH, UK; 4 Cancer Research UK | Beatson Institute, Garscube Estate, Switchback road, Glasgow G61 1BD, UK; 5 Section of Cell Biology and Physiology, Department of Biology, University of Copenhagen, Universitetsparken 13, Copenhagen DK-2100, Denmark; 6 Institut für Neuro- und Verhaltensbiologie, Universität Münster, Badestrasse 9, 48149 Münster, Germany

## Abstract

Multicellular organisms rely on cell adhesion molecules to coordinate cell–cell interactions, and to provide navigational cues during tissue formation. In *Drosophila*, Fasciclin 2 (Fas2) has been intensively studied due to its role in nervous system development and maintenance; yet, *Fas2* is most abundantly expressed in the adult renal (Malpighian) tubule rather than in neuronal tissues. The role Fas2 serves in this epithelium is unknown. Here we show that *Fas2* is essential to brush border maintenance in renal tubules of *Drosophila*. Fas2 is dynamically expressed during tubule morphogenesis, localizing to the brush border whenever the tissue is transport competent. Genetic manipulations of *Fas2* expression levels impact on both microvilli length and organization, which in turn dramatically affect stimulated rates of fluid secretion by the tissue. Consequently, we demonstrate a radically different role for this well-known cell adhesion molecule, and propose that Fas2-mediated intermicrovillar homophilic adhesion complexes help stabilize the brush border.

## Abstract (web summary)

In *Drosophila*, Fasciclin 2 (Fas2) has been mainly studied in the nervous system, yet this adhesion protein is more abundant in the adult renal tubule. Here the authors show that Fas2 is essential for brush border maintenance in renal tubules through regulation of microvilli length and organization.

## Graphical abstract

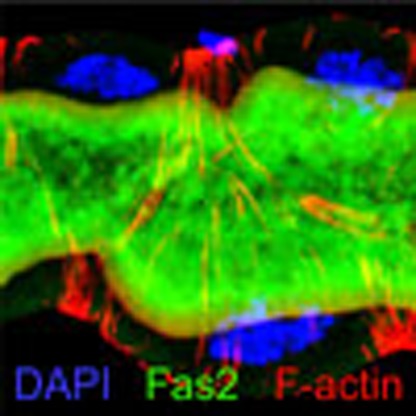

Comprehensive tissue expression atlases allow gene function to be reassessed across the whole organism^1^,^2^,^3^,^4^, and many genes show unexpected expression patterns outside the tissues in which they have been traditionally studied. One such example is Fasciclin 2 (Fas2), which has been extensively studied for its role in axon guidance and neuronal development during embryogenesis^5^,^6^,^7^,^8^. Fas2 is a cell adhesion molecule that directs axon fasciculation through homophilic cell–cell recognition, which helps to establish a regular neuronal scaffold, on which the developing nervous system is organized^5^,^9^,^10^,^11^. Surprisingly, the online resource flyatlas.org^2^,^4^ reports high postembryonic levels of expression in both larvae and adults, suggesting a lifelong function of Fas2, in addition to its well-characterized role in early development. Intriguingly, expression levels have been reported to be highest in both larval and adult renal (Malpighian) tubules^2^, yet the role Fas2 serves in this non-neuronal tissue is remarkably unexplored.

The insect Malpighian tubules (MTs) constitute the functional analogue of the vertebrate kidney, and offer a unique opportunity for the study of epithelial function and control^12^. They are composed of two morphologically and physiologically distinct cell types, the principal ‘type 1' cell and the stellate ‘type 2' cell; the larger principal cells are the sites of active cation transport, energized by an apical plasma membrane V-ATPase driving an K^+^/H^+^ exchanger, whereas the smaller stellate cells are in control of chloride and water fluxes^13^,^14^,^15^. Like all transporting epithelia, the MTs possess a prominent brush border^16^, which serves to increase the membrane surface area available to the transport machinery that drives transepithelial transport^17^. Indeed, the insect MT moves fluid faster on a per-cell basis than any other epithelium^18^. However, fundamental questions on how the MT brush border is maintained, how microvillar length is controlled and what regulates brush border organization remain unanswered. Here we report that Fas2 plays a critical role in regulating brush border length and organization in renal tubules of *Drosophila*. We show that Fas2 extracellular interactions are necessary and sufficient to mediate these effects, and that the intracellular domain is dispensable in this regard. Furthermore, we demonstrate that the transport capacity of the epithelium is correlated with Fas2-induced changes to microvillar length. Our data are thus consistent with a model in which Fas2-dependent adhesion complexes promote microvillar growth and organization, and propose that Fas2-homotypic intermicrovillar links act to stabilize the brush border against shear stress associated with the uniquely high secretion rates of the tissue.

## Results

### Transcriptomic analysis of Fas2 expression

**Figure 1:**
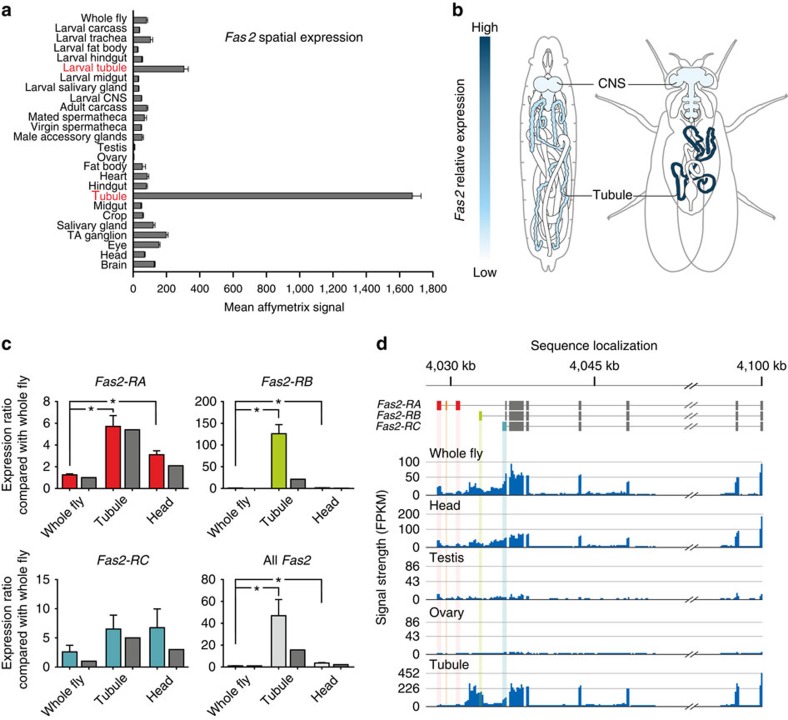
Transcriptomic profiling of *Fas2* expression. (**a**) Mean normalized Affymetrix signal±s.e.m. (*N*=4 tissue samples) showing the *Fas2* spatial expression pattern across major tissues from both larval and adult *Drosophila* (flyatlas.org). (**b**) Overview of *Drosophila* anatomy with superimposed heat maps of the spatial expression pattern of *Fas2*. (**c**) Microarray and qPCR analyses of the dominant *Fas2* isoforms across different tissues. The Affymetrix whole fly signal (dark grey bars) was set to an a.u. of 1, with head and tubule signals being expressed as a ratio to whole fly. The mean±s.d. qPCR expression ratios (light grey bars) largely reiterate the microarray data, albeit show much higher *Fas2*-*RB* transcript enrichment in MTs compared with the Affymetrix signal. *Significantly different (one-way ANOVA, *P*<0.05) compared with whole fly. (**d**) RNA-Seq data comparing *Fas2* transcript levels across different tissues. The combined transcriptomic meta-analysis reveal that *Fas2-RA*, *RB* and *RC* are the main isoforms expressed in *Drosophila*, with isoform *Fas2-RB* being the dominant splice variant expressed in the MTs.

To verify the spatial expression profile reported by FlyAtlas.org (Fig. 1a,b), and to identify the Fas2 splice variant expressed in *Drosophila* MTs, we used both quantitative reverse transcription (RT)–PCR and whole transcriptome sequencing (RNA-Seq). Both approaches independently confirmed the data reported by FlyAtlas, and further showed that although all major Fas2 isoforms are expressed, *Fas2-RB* is the dominant splice variant expressed in *Drosophila* renal tubules (Fig. 1c,d).

### Dynamic expression of Fas2 during tubule development

**Figure 2:**
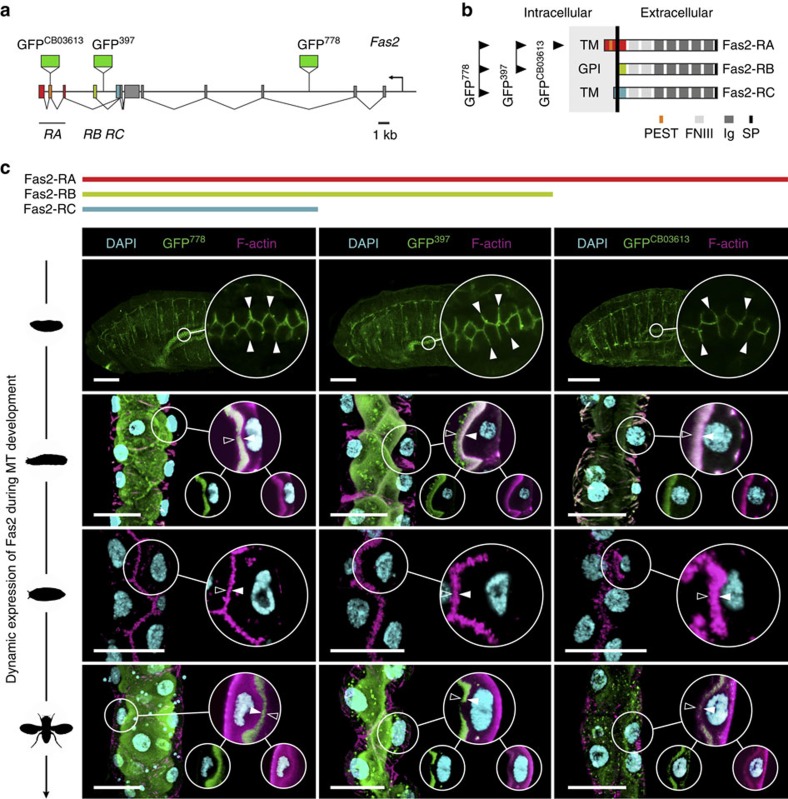
Fas2 is dynamically expressed during tubule development. (**a**) *Fas2* genomic region illustrating the positions of the GFP^CB03613^, GFP^397^ and GFP^778^ exon trap insertions. (**b**) *Fas2* is expressed in multiple isoforms that all comprise five immunoglobulin (Ig) and two fibronectin type III (FNIII) domains, yet have distinct C-termini. Arrows indicate splice variants recognized by each exon trap insertion line. The differences between transcripts *Fas2-RA, -RB* and *-RC* are highlighted in **a**,**b** using the same colour code. PEST, PEST domain; SP, signal peptide. (**c**) In stage 16 embryos, Fas2 localizes to cell junctions of the developing MTs; however, Fas2 switches abruptly to the apical brush border prior to eclosion of the first instar larva, where it remains throughout larval development. As the third instar larva pupates, the MTs become transport incompetent, the microvilli shorten, tubule lumen is reduced and Fas2 is no longer expressed. As the brush border reform in the adult tubule, Fas2 is again abundantly expressed in the MT, where it localizes to the apical brush border (area between arrows). This expression pattern is consistently reported by each fusion protein, and is commensurate with the transcription profile of each *Fas2* isoform. Inserts: single optical section of the selected region shown as separate (small circle) and merged (large circle) signals. Scale bars, 25 μm.

To gain insight into what role Fas2 serves in MTs of *Drosophila*, we initially identified the subcellular localization of Fas2 protein using genetic and immunocytochemical approaches. During embryogenesis, Fas2 is known to localize to the lateral junctions of the developing MTs^19^, which is consistent with its role in cell–cell recognition in the central nervous system (CNS)^5^. Yet, whether this localization pattern remains static throughout tubule development is unknown. We therefore investigated the spatio-temporal expression pattern of Fas2 during tubule morphogenesis, by utilizing a series of Fas2 exon trap insertion lines that recognize different Fas2 isoforms (Fig. 2a,b). Each fusion protein—known to behave as native protein in the CNS^20^,^21^—reported the same distribution pattern (Fig. 2c): Fas2 localizes to the lateral cell junctions in MTs of late stage embryos (stage 16<), but abruptly switches to the apical brush border (area between arrows; Fig. 2c) of the principal cells during early larval development, where it remains until pupation. In the transport incompetent pupal tubules—evident by the involuted brush border and reduced tubule lumen^22^—Fas2 completely vanishes, then reappears as the adult microvilli form and remains throughout adulthood (Fig. 2c). The observed changes in brush border appearance during tubule development (see Fig. 6a) is consistent with that previously described at an ultrastructural level^22^. The localization of Fas2 to the apical brush border was confirmed by immunocytochemistry using two separate anti-Fas2 monoclonal antibodies (Supplementary Fig. 1). These data thus show a highly dynamic expression pattern of Fas2 during tubule development, and indicate that MT transport competence partly depends on Fas2 localization to the brush border.

### Fas2 localizes extracellularly between microvilli

**Figure 3:**
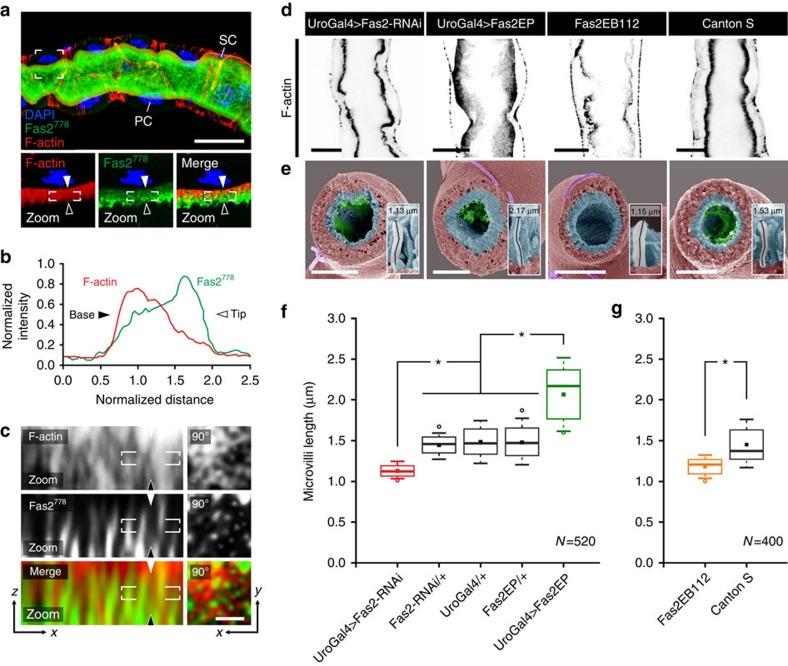
Genetic manipulation of *Fas2* impacts microvilli length. (**a**) Super-resolution confocal microscopy (Airyscan) on MT from Fas2–GFP^778^ stained with anti-GFP, suggests that Fas2 localizes to the brush border, where it appears concentrated distally. Zoom: single optical section of the indicated region shown as separate and merged signals. Arrows indicate base (solid) and tip (line) orientations of the brush border. PC, principal cell; SC, stellate cell. Scale bar, 25 μm. (**b**) Mean normalized fluorescent intensity profiles for F-actin and Fas2 signals from *N*=12 brush border regions confirm that Fas2 is concentrated at the distal tip of the F-actin-based protrusions. (**c**) Magnification of the white square in ‘**a** zoom', and a perpendicular view (‘90°') on the brush border, suggests that Fas2 does not strictly colocalize with intracellular F-actin (phalloidin), but is positioned extracellularly between individual microvilli. Scale bar, 0.5 μm. (**d**) Inverse coloured optical sections of Alexa-488-phalloidin (black) stained MTs from Fas2 knockdown flies (Fas2-RNAi and Fas2EB112) showed a marked decrease in length and density of the microvillar brush border compared with WT (Canton S) tubules. Conversely, overexpression of Fas2 (Fas2-EP) produced notably longer and denser microvilli. Scale bars, 15 μm. (**e**) Scanning electron microscopy (SEM) analysis of MT cross-sections (brush border pseudo-coloured in cyan) from adult *Drosophila* using the principal cell-specific UroGAL4 driver to drive both RNAi and overexpressor constructs confirmed these results. Individual microvilli were measured (see inserts) from (*N*=10–13) cross-sections with (*N*=400–520) microvilli measured in total for each *Fas2* genetic background. Scale bars, 20 μm. (**f**,**g**) Tukey box and whisker plots of microvilli length from the different *Fas2* genetic backgrounds. Genetic manipulations of *Fas2* expression levels significantly reduced or increased (*, one-way ANOVA, *P*<0.05) microvilli length compared with parentals or WT. Solid squares indicate mean values; open circles symbolize data outliers.

As Fas2 has been shown to be a key mediator of axon fasciculation through homophilic adhesion^5^,^7^,^21^, we rationalized that Fas2 might be involved in the structural stabilization of microvilli, by forming homotypic intermicrovillar links that help to maintain their ‘fasciculation'. To test this hypothesis, we performed super-resolution confocal microscopy. This revealed that although Fas2 colocalizes with F-actin throughout the proximal-distal axis of the brush border concentrating distally (Fig. 3a,b), the Fas2 signal appears to predominantly interpolate between discrete F-actin signals (Fig. 3c). This is consistent with F-actin microfilaments marking the intracellular region of the microvilli, and the GFP^778^ tag labelling the extracellular domain of Fas2 (Fig. 2a,b), resulting in a limited overlap between signals. These findings indicate that Fas2 localizes extracellularly, and is properly positioned to contribute to intermicrovillar links.

### *Fas2* expression affects microvilli length and organization

**Figure 4:**
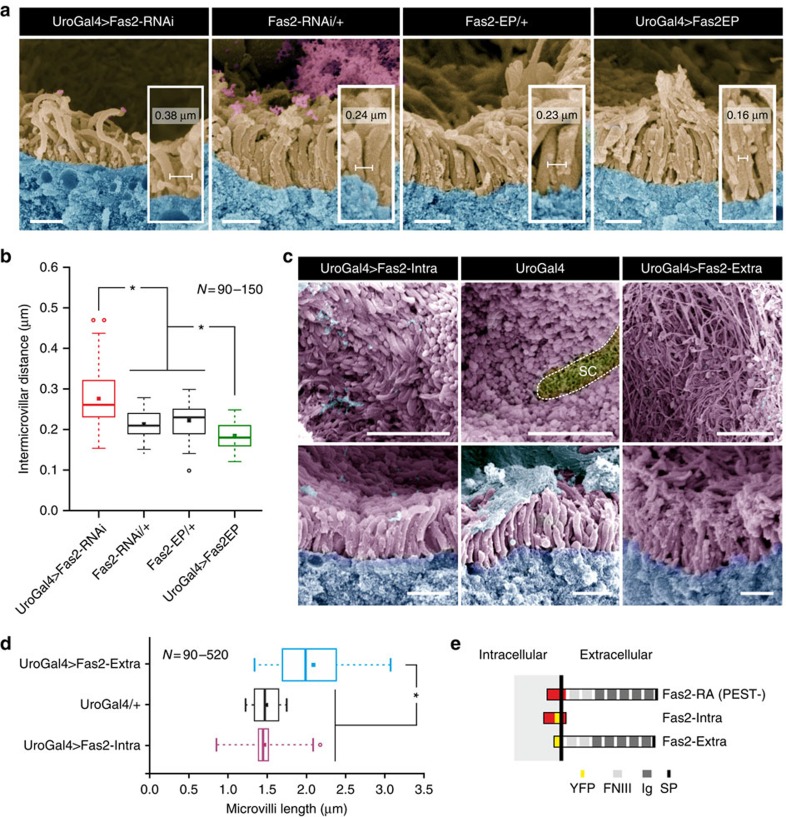
Genetic manipulation of *Fas2* impacts microvilli organization. (**a**) Scanning electron microscopy (SEM) analysis of microvillar organization. Microvilli (pseudo-coloured light brown) from parentals (Fas2-RNAi/+ and Fas2-EP/+) show distinct well-organized bundles of microvilli, whereas in *Fas2* knockdown flies (UroGAL4>Fas2-RNAi) the microvilli tend to appear as single distinct protrusions. Overexpressing *Fas2* makes the bundles more obvious, and the microvilli often appearing more densely packed (UroGAL4>Fas2-EP). Inter-microvilli distances were measured (see inserts) from (*N*=6–10) cross-sections with (*N*=90–150) microvilli measured in total for each *Fas2* genetic background. Scale bars, 1 μm. (**b**) Tukey box and whisker plot of intermicrovillar distances from the different *fas2* genetic backgrounds. Genetic manipulations of *Fas2* expression levels significantly change (*, one-way ANOVA, *P*<0.05) the average distance between the individual microvillar protrusions compared with parental controls. Intermicrovillar distance was measured (see inserts) from (*N*=6–10) cross-sections with (*N*=90–150) distances measured in total for each *Fas2* genetic background. Solid squares indicate mean values; open circles symbolize data outliers. (**c**) Independent overexpression of the Fas2 extracellular domain (Fas2-Extra–YFP) results in a phenotype comparable to overexpressing the full-length transcript (although areas of the brush border showed an excess of adhesion-activity compared with parental controls), whereas overexpression of the Fas2 intracellular domain (Fas2-Intra–YFP) results in microvilli being indistinguishable from controls. Top tier images represent face-on views, whereas bottom tier images are cross-sectional views of the brush border from the various genetic backgrounds. SC, stellate cell (yellow). Scale bars, 5 μm and 1 μm, respectively. (**d**) Tukey box and whisker plot of microvilli length from the selective overexpression of the Fas2 extracellular and intracellular domains, respectively. Overexpression of the extracellular domain significantly changes (*, one-way ANOVA, *P*<0.05) the average microvillar length compared with overexpression of the intracellular domain and parental controls. Individual microvilli were measured from (*N*=5–10) cross-sections with (*N*=90–520) microvilli measured in total for each *Fas2* genetic background. Solid squares indicate mean values; open circles symbolize data outliers. (**e**) Schematic diagram of native full-length Fas2-RA (PEST-) and the YFP-tagged Fas2-Intra and Fas2-Extra constructs (adapted from ref. 7).

To gain further insight into the functional role of Fas2 in renal tubules, we restrictively modulated expression levels of *Fas2* by driving either RNAi or overexpressor constructs in the principal cells with the GAL4/UAS system^23^,^24^. Confocal microscopy of tubules stained with phalloidin (F-actin) confirms that wild type (WT) tubules have a strict organized, dense population of microvilli at the brush border of the principal cells (Canton S, Fig. 3d). On significant reduction (one-way analysis of variance (ANOVA), *P*<0.05) of Fas2 transcript and protein levels (Supplementary Fig. 2), the brush border becomes sparse, shorter and less organized (Fas2-RNAi and Fas2EB112, Fig. 3d). Conversely, increased levels of Fas2 result in the opposite phenotype, where the brush border appears denser and longer (Fas2-EP, Fig. 3d). Scanning electron microscopy on tubules from the same genetic backgrounds (Fig. 3e) confirmed these results, and additionally allowed quantification of microvilli length, which demonstrated that *Fas2* depletion (UroGAL4>Fas2-RNAi and Fas2EB112) results in a significant decrease in microvilli length compared with both parental lines (Fas2-RNAi/+, UroGal4/+ and Fas2-EP/+) and WT (Canton S), respectively (Fig. 3e–g). Conversely, increased levels of *Fas2* cause a significant increase in microvilli length compared with controls (Fig. 3e,f); these findings were reiterated using two separate principal cell-specific GAL4 drivers (Supplementary Fig. 3). Further analysis of the microvillar protrusions indicated that the organization (in addition to the length) of the microvilli also was disrupted on manipulation of Fas2 levels (Fig. 4a,b; Supplementary Fig. 4). Control tubules (Fas2-RNAi/+ and Fas2-EP/+) have a highly organized structure of microvilli with a tendency for small, well-distributed clusters. However, decreased levels of Fas2 (UroGAL4>Fas2-RNAi) resulted in less dense and more disorganized microvilli (Fig. 4a; Supplementary Fig. 4), whereas overexpression of Fas2 (UroGAL4>Fas2-EP) frequently gave rise to both larger and longer bundles of microvilli (Fig. 4a; Supplementary Fig. 4). Moreover, there is a significant increase in the distance between individual microvilli when Fas2 is decreased, and a slight, but significant, decrease in intermicrovillar distance when Fas2 levels are increased (Fig. 4b). Taken together these results indicate that endogenous levels of Fas2 are required for the development of microvillar protrusions with stereotypic length and organization.

### Genetic dissection of Fas2 function

All Fas2 isoforms contain an extracellular domain with multiple Ig-binding domains; yet the isoforms differ in their membrane-anchoring or intracellular regions (Fig. 2a,b). In the developing CNS, Fas2-RA is capable of signalling intracellularly through its cytoplasmic domain^5^. In contrast, the Fas2-RB isoform is predicted to lack a transmembrane region^25^, and instead link to the membrane via a GPI anchor^26^. Because the MTs express all three major splice variants (Fig. 1c,d), the microvillar stabilization observed could result from either intracellular signalling to the cytoskeleton, direct homophilic binding between microvilli or both. As the tubule shows highest expression of the *Fas2-RB* transcript (Fig. 1c,d), we rationalized that homotypic binding of ectodomains acts to stabilize microvillar length by forming extracellular links of Fas2, analogous to the intercellular homophilic interactions demonstrated in the developing CNS and neuromuscular junctions^5^,^7^. To test this model, we selectively overexpressed either the extracellular or intracellular domains in tubule principal cells (Fig. 4c–e). When comparing tubules with endogenous levels of Fas2 (UroGAL4), overexpression of only the extracellular domain (UroGAL4>Fas2-Extra) resulted in longer microvilli, comparable to the overexpression phenotype of the full-length transcript (Figs. 3f, 4d). However, overexpression of the extracellular domain (UroGAL4>Fas2-Extra) also resulted in areas of the brush border, demonstrating aberrant microvilli organization compared with parental controls (UroGAL4; Fig. 4c), which may suggest that the GPI anchor of the full-length protein is important for controlling how the brush border is organized. In contrast, when only the intracellular domain is overexpressed (UroGAL4>Fas2-Intra), the microvilli are indistinguishable from those from control tubules (Fig. 4c), indicating that the intracellular domain, of the less abundant *Fas2-RA* splice variant, is not involved in regulating microvilli length or organization (Fig. 4d). Together, these observations support the hypothesis that Fas2 extracellular domains are necessary to stabilize the brush border.

### Microvilli length correlates with MT transport capacity

**Figure 5:**
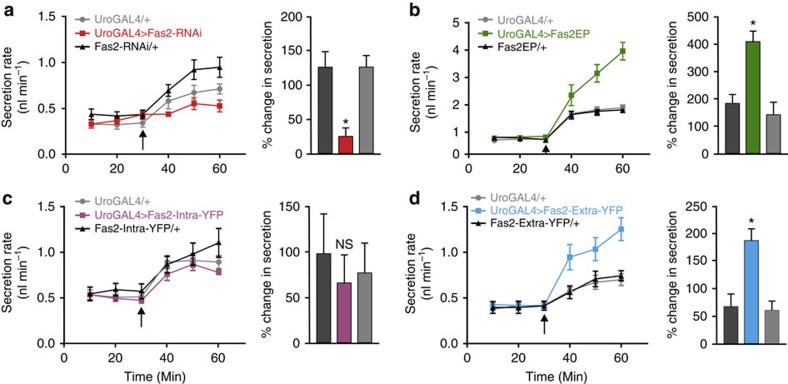
Genetic manipulation of *Fas2* impacts renal function. (**a**) Knock down (red) of *Fas2* results in a significant reduction, while (**b**) overexpression (green) of *Fas2* causes a significant increase, in cAMP-stimulated rates of fluid secretion compared with parental controls (*, one-way ANOVA, *P*<0.05). (**c**) Overexpression of only the Fas2 intracellular domain (magenta) has no significant effect on the cAMP-induced secretion rates (NS, one-way ANOVA, *P*<0.05), while (**d**) overexpression of the extracellular domain alone (blue) significantly increased (*, one-way ANOVA, *P*<0.05) stimulated fluid secretion rates compared with parental lines. Arrows indicate point of cAMP (10^−4^ M) addition. Data are expressed as mean±s.e.m. of *N*=6–7.

Microvilli are classically observed on the apical surface of transporting epithelia, where they are thought to increase the membrane area available for transmembrane transport^27^,^28^. Consistent with this, when microvilli are compromised, for example, in coeliac disease or in Usher syndrome, malabsorption and/or renal tubular dysfunction results^29^,^30^. Manipulation of *Fas2* levels allows direct testing of this model; if the longer microvilli in *Fas2* overexpressors are physiologically functional, these tubules should be able to secrete fluid at a higher rate. This can be quantitatively tested in *Drosophila*, by measuring fluid secretion rates^12^ both at rest and under stimulation. Tubules actively transport cyclic nucleotides, and so can be stimulated by addition of extracellular cyclic AMP (cAMP)^12^,^31^. Accordingly, secretion rates in control tubules are clearly elevated on cAMP addition (Fig. 5a–d). Although basal secretion rates are unaffected by manipulation of *Fas2* expression—the utility of the increased membrane area is not exploited unless the tissue is stimulated—knockdown of *Fas2* (red) resulted in a significantly reduced (*, one-way ANOVA, *P*<0.05) stimulated rate of secretion (Fig. 5a). Conversely, overexpression of either the full-length (green) or extracellular domain (blue) of *Fas2* significantly increased (*, one-way ANOVA, *P*<0.05) the stimulated response (Fig. 5b–d); the apparent discrepancy in brush border organization between overexpressing the full-length and the extracellular domain, respectively (Fig. 4a,c), likely explains the observed difference in the maximal performances of the tubules from the two genotypes (Fig. 5b–d). By contrast, overexpression of just the intracellular domain (magenta) had no effect (Fig. 5c). Together, these results indicate that the transport capacity of the tissue is proportional to Fas2-induced changes to brush border size, and that the increased membrane area is functionally significant.

## Discussion

**Figure 6:**
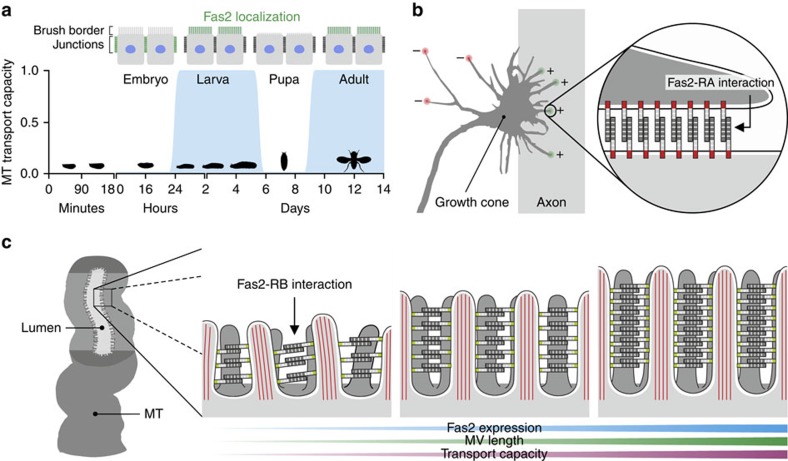
Hypothesis for Fas2 function in the *Drosophila* MT. (**a**) Schematic representation of the spatio-temporal expression pattern of Fas2 (green) during tubule development. Fas2 does not localize to the microvilli when the tubules are transport incompetent, but are abruptly recruited to the microvillar brush border when the tissue develops transport competence (blue). (**b**) Schematic representation of how Fas2 is proposed to provide attractive forces through homophilic adhesion on the axon relative to the environment that promotes axon fasciculation and growth cone guidance (based on ref. 11). (**c**) Proposed model for Fas2 function in the adult *Drosophila* MT. By contrast to the *Drosophila* nervous system and neuromuscular junction, where Fas2 mediates intercellular interactions, we hypothesize that Fas2 acts as homotypic bridging protein within the same cell of the renal tubule, where it stabilizes the microvillar brush border against shear stress caused by uniquely high flux rates. Knockdown of Fas2 results in shorter and disorganized microvilli with the opposite being the case when Fas2 is overexpressed.

How microvillar protrusions are assembled and maintained in different epithelial tissues is still largely unknown. In this study, we show that the cell adhesion molecule Fas2 is dynamically expressed during MT development—localizing to the brush border of transport competent renal tubules (Fig. 6a)—and demonstrate that tissue-specific genetic manipulation of Fas2 expression levels elicits concentration-dependent effects on microvillus length and brush border organization (Figs 3 and 4). Similar observations have been made on other cell adhesion molecules in both vertebrate^32^,^33^ and invertebrate^34^,^35^ epithelial systems, which suggests that cell adhesion molecules likely play a universal role in regulating the assembly and maintenance of actin-based cellular protrusions. Furthermore, the apparent diversity of cell adhesion molecules recruited to perform seemingly identical tasks in different epithelia (for example, Cad99C in *Drosophila* follicle cells^34^,^35^ and Fas2 in *Drosophila* tubule cells) indicates that different adhesion molecules likely help define structural properties specific to individual tissues.

In *Drosophila* follicle cells^34^,^35^, as well as in the vertebrate inner ear and intestinal epithelia^29^,^32^, members of the cadherin superfamily of cell adhesion molecules have been shown to contribute to heterophilic adhesion complexes between adjacent F-actin-based protrusions. In contrast, Fas2 has been demonstrated to be capable of forming homophilic complexes in both *in vitro*^5^ and *in vivo*^7^ studies (Fig. 6b). As our data suggest Fas2 is positioned extracellularly between individual F-actin bundles (Fig. 3a–c), it seems highly likely that Fas2 is involved in forming homotypic intermicrovillar links in the *Drosophila* MT (Fig. 6c). Through genetic dissection of Fas2 function, we further found that membrane-anchored Fas2 ectodomains are sufficient to mediate the observed changes in brush border length and integrity, and that the cytoplasmic domain of the less abundant *Fas2-RA* isoform is dispensable (Fig. 4). These observations are strongly mirrored by the molecular function of Cad99C in *Drosophila* follicle cells^34^,^35^; a protein that is also known to interact with the unconventional motor protein Myosin VIIA (Myo7A), to further control the length and organization of microvilli^36^. Interestingly, both Cad99C and Myo7A, according to flyatlas.org^2^, are most highly enriched in larval and adult MTs, respectively, indicating that these proteins might similarly play a role in brush border assembly and/or maintenance in *Drosophila* renal tubules. Yet, irrespective of the binding partners involved, how might adhesion complexes control microvilli length? The lengthening or shortening of microvilli actin-core filaments depends on the assembly versus disassembly rates of actin polymerization, through a process called treadmilling^37^. According to this model, the force created by net-positive treadmilling rates has to overcome the opposite force generated by tension of the plasma membrane as the actin-based protrusion extends. As such, stabilization of the plasma membrane envelope by intermicrovillar links might help alleviate some of this tension, and thus promote actin-core filament growth and microvilli extension^34^. Alternatively, the length of microvilli protrusion in insect MTs could be physically constrained by the shear stress caused by high luminal flux rates, in which case a scaffold composed of Fas2-homotypic links might help drive actin-core assembly/disassembly dynamics towards microvilli elongation. Both models—which are not mutually exclusive—could explain the correlation between microvilli length and organization and Fas2 expression levels. Whether additional factors are involved in regulating this mechanism, for example, targeting Fas2 to the brush border, anchoring Fas2 to the actin core, or regulating Fas2 adhesive activity—as demonstrated for other ‘scaffolding' proteins^30^,^31^,^32^,^33^,^34^,^35^,^36^—remains unknown.

Based on our collective findings, we propose a model for Fas2 function, in which Fas2 homophilic binding between adjacent microvilli forms intermicrovillar links, which help stabilize the brush border against shear stress (Fig. 6c). As argued, this model is both a logical extension of what is known about the Fas2 cell adhesion properties^5^,^7^ and insect renal tubule physiology^18^, and is furthermore consistent with the concentration-dependent effect observed between Fas2 activity on microvilli length and organization (Figs 3 and 4). Our study thus provides a radically new view on the function, outside the CNS, of one of the best-known neural development molecules, and moreover suggests that while actin involvement is known to be critical for microvillar formation, the molecular machinery responsible for brush border assembly and maintenance in different epithelial systems is far from understood.

## Methods

### Fly strains

All fly lines were cultured on standard medium over a 12:12 h photoperiod at 45–55% humidity at 22 °C. Ectopic gene expression using the GAL4/UAS system was carried out at 26 °C. The *Fas2* exon trap insertions *Fas2*^*GFP397*^ and *Fas2*^*GFP778*^ (this study) were isolated in exon trap screens^21^, while *Fas2*^*GFPCB03613*^ was obtained from the Spradling lab^38^. The UAS*Fas2-Intra–YFP* and UAS*Fas2-Extra–YFP* lines were a kind gift from the Nose lab^7^, whereas the *Fas2*^*EB112*^ hypomorph was obtained from the Goodman lab^5^. The Fas2 overexpressor *Fas2EP*^39^ was obtained from the Bloomington Stock Center, and the *Fas2RNAi* line was acquired from the Vienna *Drosophila* RNAi Centre (VDRC). The UroGAL4 (ref. 40) and CapaRGAL4 (ref. 41) drivers, both specific to principal cells of MTs, were developed in-house and used to drive all constructs.

### Quantitative RT–PCR

Fas2 expression levels were verified using qPCR. A two-step qPCR was carried out using the fluorescent double-stranded DNA dye DyNAmo SYBR Green (Finnzymes, Finland). Prior to carrying out these experiments, complementary DNA was synthesized from the tissue of interest using SuperScript IV (Invitrogen, CA, USA). For each experiment, four biological replicates were generated, and three technical replicates were loaded for each. Primers were designed to produce products <500 bp, and where possible, to span exon/intron boundaries of the gene of interest. Primers were also designed against the housekeeping gene alpha-tubulin, to normalize samples (forward: 5′- AGGGTATGGAGGAGGGAGAGTTC -3′; reverse: 5′- TGCGATTGGAAGCGTAAACAC -3′). Template amplicons were generated for each primer pair, and standards ranging from 10^−1^ to 10^−7^ ng were created by serial dilution, allowing for absolute quantification of gene expression. Cycling was performed in Opticon 3 thermal cycler (BioRad, UK). Following amplification, Opticon 3 software was used to generate a standard curve. Relative concentration was determined by placing the Cycle Threshold (Ct) value and the values from the gene standard onto the standard curve. Each sample was then normalized against alpha-tubulin, resulting in a ratio of gene/alpha-tubulin expression. Results were then plotted as means±s.e.m. (where control=1) using Prism 6.0 (GraphPad, CA, USA).

### Whole transcriptome shotgun sequencing (RNA-Seq)

The *Fas2* gene is predicted to be differentially spliced into multiple isoforms (Fas2-RA to Fas2-RH; Metazoa.Ensembl.org) of which only Fas2-RA (+/− PEST), -RB and -RC have been experimentally confirmed^21^. To identify which isoforms of Fas2 are expressed in MTs of *Drosophila*, we therefore performed RNA-Seq. In brief, MTs were dissected on ice, and total RNA was extracted using a Qiagen RNAeasy kit. RNA library preparation was performed using directional RNA (polyA selection) Illumina library preparation kit according to manufacturer's protocol, and samples were subsequently run on a Solexa Genome Analyzer II RNA-Seq system with 10M paired-end reads (75 bp). The resultant FASTQ files were processed using the open-source software tools TopHat and Cufflinks^42^, using the latest version of the *Drosophila* reference genome sequence^43^.

### Immunocytochemistry

For embryo collection, adult females were allowed to lay eggs on grape juice agar plates for ∼16–24 h at 26 °C. Embryos were next collected and dechorionated in a fresh solution of 50:50 bleach (10%) and ddH_2_O for exactly 3 min, and then washed thoroughly with ddH_2_O. Using a fine brush, the embryos were subsequently transferred to 5 ml of heptane (embryos should sink), and 5 ml of 4% PFA in PBS was added, before shaking the liquid to saturate the mixture. Samples were left for 30 min on a shaker to fix. The lower aqueous phase was then gently removed, and 5 ml of a fresh solution of 9:1 MeOH (100%) and 50 mM EGTA (pH 8) was added, and the sample gently shaken for 3–5 min. Devitellinized embryos, which appeared in the MeOH/EGTA phase, were collected and placed in PBS before being mounted and imaged. In contrast, acutely dissected MTs from both larvae and adults, were fixed in 4% paraformaldehyde in PBS for 12 min, and incubated in PBS with 10% NGS and 0.1% Triton X-100 (PBT) and rabbit-anti-green fluorescent protein (GFP) conjugated to Alexa Fluor 488 (1:200; Molecular Probes, OR, USA, cat# A21311) where necessary. Alternatively, fixed tissues were incubated overnight in PBT with either mouse anti-Fas2 1D4 (1:80; DSHB, deposited by Goodman) or 34B3 (1:40; DSHB). Following several washes in PBT, the tissues were then incubated with Alexa Fluor 488-conjugated goat-anti-mouse secondary antibody (1:200; Thermo Fisher Scientific, MA, USA, cat# A-10667), DAPI (4′,6-diamidino-2-phenylindole, 1 μg ml^−1^; Thermo Fisher Scientific, cat# D1306) and Rhodamine-coupled phalloidin (1:100; Thermo Fisher Scientific, cat# R415) in PBT for a minimum of 2 h. Next, samples were washed repeatedly in PBS before being mounted on poly-L-Lysine (0.1% w/v in H_2_O, Sigma-Aldrich, MO, USA) covered 35 mm glass bottom dishes (MatTek Corporation, MA, USA) in Vectashield (Vector Laboratories Inc, CA, USA) and image acquisition was performed on an inverted Zeiss LSM 510 Meta confocal microscope (Zeiss, Oberkochen, Germany).

### Super-resolution microscopy

To improve visualization of the subcellular localization of Fas2, we performed super-resolution microscopy using a Zeiss LSM 880 inverted confocal microscope equipped with Airyscan (kindly lent by Zeiss), providing an optical resolution of ∼140 nm. Tissue samples were prepared as described above. Line scans and normalized fluorescent intensity profiles of the F-actin and Fas2 signals across the MT brush border were generated using the ZEN blue software (Zeiss).

### Scanning electron microscopy

MTs were dissected from 7-day-old flies (males and females) under Schneider's insect medium (Invitrogen), and subsequently fixed in 2.5% glutaraldehyde in 0.1 M cacodylate buffer (pH 7.4) for 90 min^15^,^44^. The tissue was then rinsed repeatedly in ddH_2_O before being dehydrated through a graded ethanol series, and desiccated using an Autosamdri-815 critical point dryer (Tousimis Research Corporation, Maryland, USA). The tubules were then transferred to aluminium stubs, fractioned and coated with platinum (70 s ∼12 nm thickness) in a JEOL JFC-2300HR high-resolution fine coater (Jeol, Tokyo, Japan) and examined with a JEOL JSM-6335-F scanning electron microscope (Jeol). Quantification of microvilli length (μm) and intermicrovillar distance (μm) was done post image acquisition for each relevant genotype. Length was measured from base to tip of 40 microvilli from each cross-section, with 10–13 cross-sections providing the mean microvilli length for each genotype. The distance between individual microvilli was measured from the centre of each adjacent microvillus with minimum 15 intermicrovillar distances measured per cross-section and a total of 6–10 cross-sections from each genotype. All measurements were performed exclusively on microvilli in which these metrics were clearly discernable (see Fig. 3e, inserts; Fig. 4a, inserts) and only on principal ‘type I' cells of the main segment^22^.

### Ramsay fluid secretion assay

Intact MTs were dissected from native tissue and set up as *in vitro* preparations by isolating them in drops of a freshly prepared mixture of Schneider's medium and insect saline (1:1, v/v) under water-saturated liquid paraffin, with one end being wrapped around a metal pin and the other end submerged in the saline drop^12^,^15^. Secreted fluid would then accumulate as a discrete droplet from the common ureter. A drop of secreted fluid was subsequently collected at appropriate intervals, and the diameter measured using an eyepiece graticule. The volume of each droplet was calculated as (4/3)*πr*^3^, where *r* is the radius of the droplet, and secretion rates plotted against time. Secretion was measured under basal conditions to establish a steady rate of secretion, prior to stimulation with 10^−4^ M cAMP. Per cent change from basal rates of fluid secretion immediately prior to cAMP application, compared with 30 min after stimulation, was statistically tested between the parentals and the different *Fas2* genetic backgrounds.

### Western blotting

For each genetic background, MTs from >70 flies were dissected under Schneider's medium and subsequently transferred to 100 μl RIPA buffer (150 mM NaCl, 10 mM TrisHCL ph 7.5, 1 mM EDTA, 1% Triton X-100, 0.1% SDS) with HALT protease inhibitor (Thermo Fisher Scientific). Samples were homogenized using a Microson XL2000 sonicator (Misonix Inc. NY, USA), and ultra centrifuged (17,000*g*) at 4 °C for 5 min, before transferring the supernatant to fresh tubes. Protein contents were measured using the Bradford assay (Precision Red, Cytoskeleton). Approximately 15 μg protein from each sample was electrophoresed on a NuPage 4–12% Bis-Tris gel (Invitrogen), and blotted onto nitrocellulose using the Novex system (Invitrogen). Blots were probed with monoclonal anti-Fas2 34B3 (1:500) and anti-GAPDH (1:1,000, Abcam, UK, cat# ab125247) antibodies, and developed with DyLight800-conjugated goat anti-mouse IgG (1:10,000, Thermo Fisher Scientific, cat# SA5-35521) and Alexa Fluor 680-conjugated goat anti-rabbit IgG (1:10,000, Thermo Fisher Scientific, cat# A-21109) secondary antibodies, using a Li-Cor CLx Odyssey. Fas2 expression was quantified relative to GAPDH levels using Image Studio Lite software (Li-Cor Biosciences), and the data represented as mean±s.e.m. based on three independent experimental repeats. The full scan is provided in Supplementary Fig. 5.

### Statistics

Data were tested using one-way ANOVA followed by Tukey's multiple comparisons of means with a significance level of *P*<0.05 (*significant; not significant (NS)). The statistical tests were performed using the data analysis programs OriginPro 7.5 (OriginLab, MA, USA) or Prism 6.0.

## Additional information

**How to cite this article:** Halberg, K. A. *et al*. The cell adhesion molecule Fasciclin2 regulates brush border length and organization in *Drosophila* renal tubules. *Nat. Commun.* 7:11266 doi: 10.1038/ncomms11266 (2016).

## Supplementary Material

Supplementary Information Supplementary Figures 1-5
