# Supplementary material for: The cell adhesion molecule Fasciclin2 regulates brush border length and organization in Drosophila renal tubules
Source: Nat Commun. 2016 Apr 13;7:11266. doi: 10.1038/ncomms11266 (PMC4833865; doi:10.1038/ncomms11266)
Supplement: Supplementary Information — Supplementary Figures 1-5 [file ncomms11266-s1.pdf]

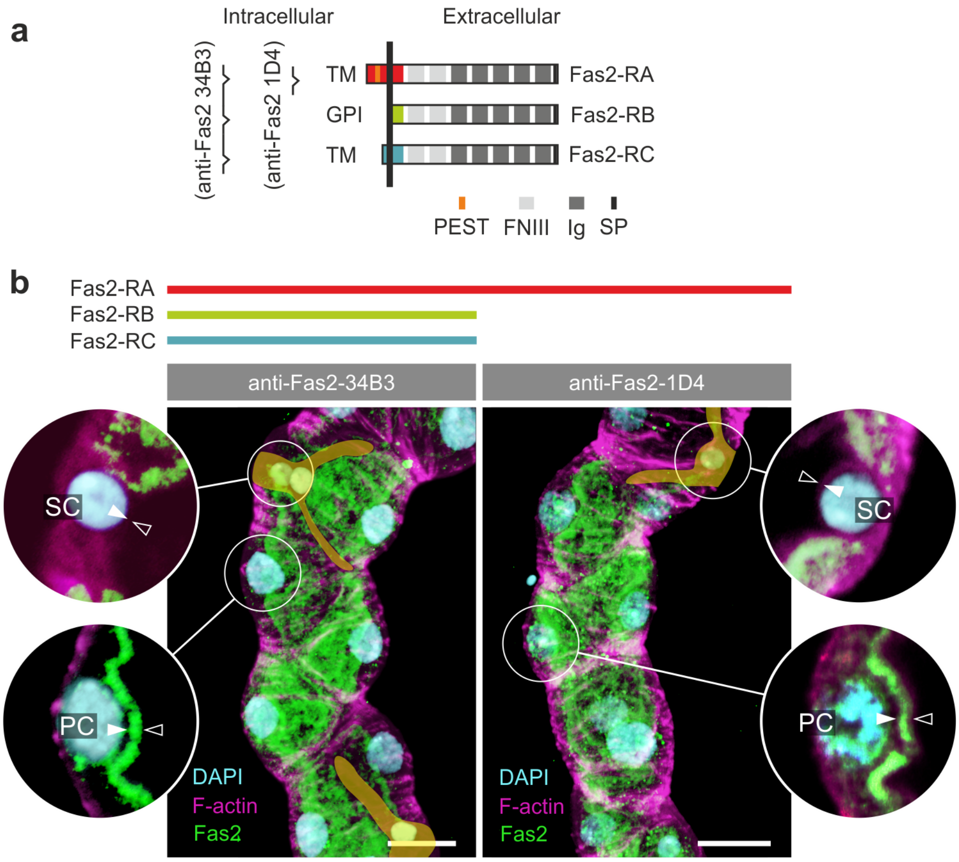

Supplementary Figure 1 | Immunocytochemical localization of Fas2. (a) Schematic representation of the dominant Fas2 isoforms and their functional domains. The Fas2 isoforms recognized by each anti-Fas2 antibody is indicated by arrows. SP, signal peptide; PEST, PEST domain. **(b)** Fas2 is shown to localize to the apical brush border (arrows) of principal cells (PC), but not stellate cells (SC), of adult WT (Canton S) MTs using the monoclonal anti-Fas2 antibodies 1D4 and 34B3. SCs are marked in yellow. This is consistent with that reported by the *Fas2* protein trap insertion lines. Inserts represent single optical sections of the selected regions. Scale bars, 25 μm.

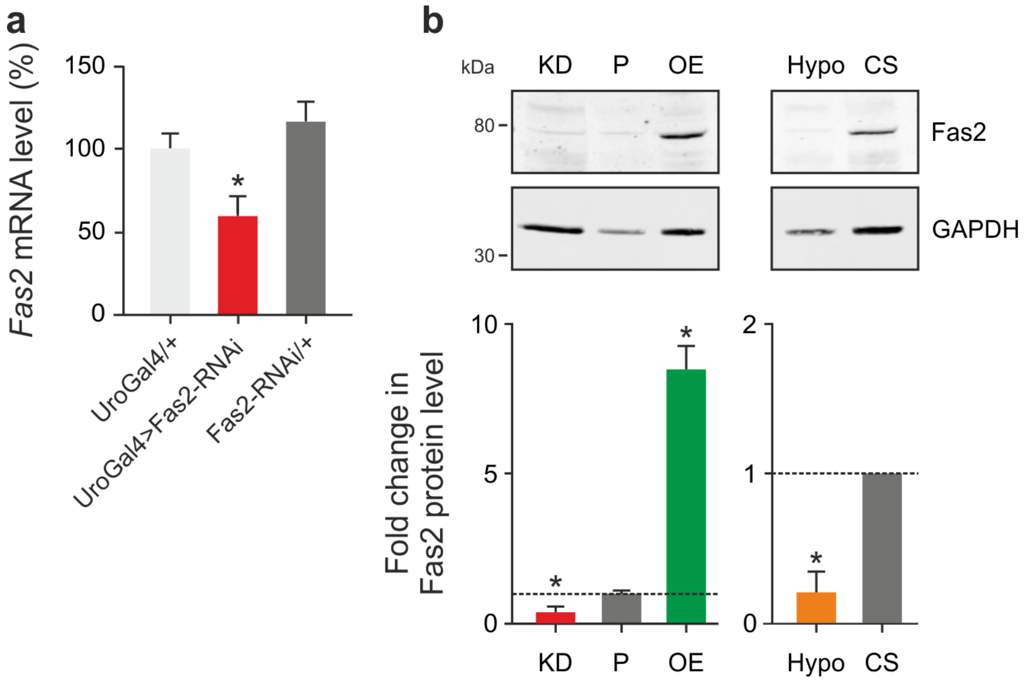

Supplementary Figure 2 | Validation of *Fas2* expression and protein levels. (a) Expression of *Fas2* mRNA levels was normalized against the housekeeping gene *alpha-tubulin*, and values expressed as mean percent change ± s.e.m. compared to parental controls (*N*=3). The observed difference in sample quantity corresponds to >40% knockdown, which is significantly different (*, one-way ANOVA, *P*<0.05) from both parental controls. **(b)** Western blot analysis confirmed these results on a protein level, further showing a >0.6 fold knockdown (KD, UroGal4>Fas2-RNAi) and a >8 fold increase (OE, UroGal4>Fas2-EP) in Fas2 protein levels compared to parental (P, UroGal4). Furthermore, the hypomorph (Hypo, Fas2-EB^{112}) showed a >0.8 fold decrease in Fas2 protein compared to Canton S (CS). Fas2 protein levels were normalized against the housekeeping protein GAPDH, and values expressed as fold change relative to either parental (P) or Canton S (CS).

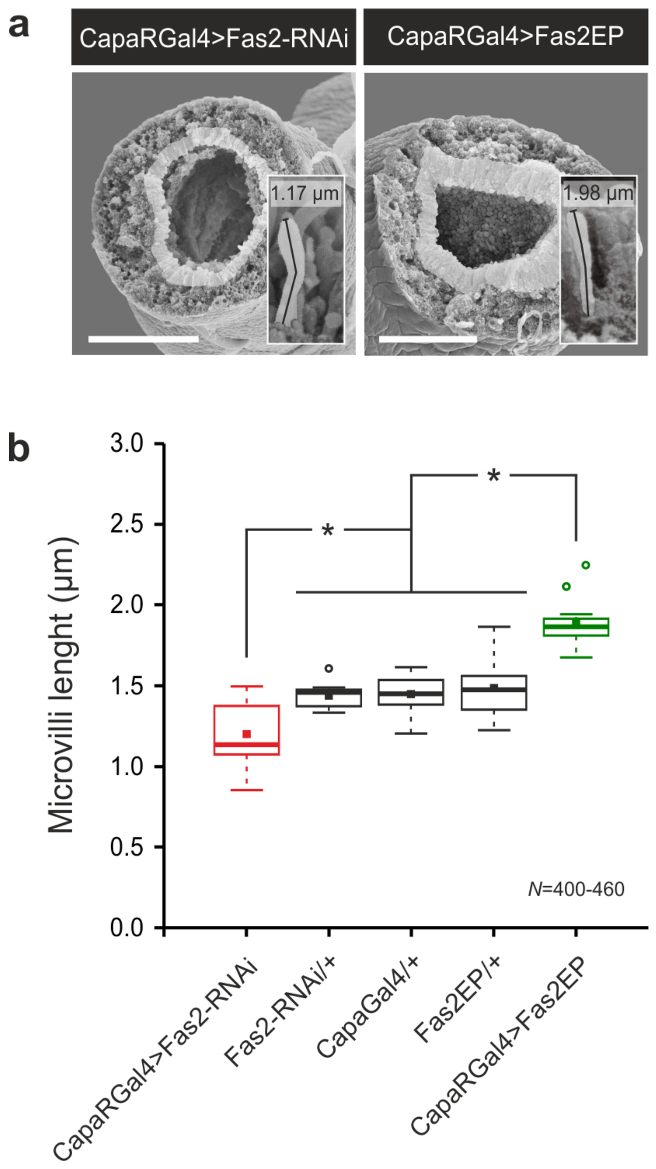

Supplementary Figure 3 | Genetic manipulation of *Fas2* expression impacts microvilli length. (a) SEM analysis of MT cross-sections (main segment) from adult *Drosophila* using the principal cell specific CapaRGal4 driver to drive both RNAi and overexpressor constructs. Individual microvilli were measured (see insert) from (*N*=10-13) cross-sections with (*N*=400-460) microvilli measured in total for each *Fas2* genetic background. Scale bars, 20 µm. **(b)** Tukey boxplots of microvilli length from the different *Fas2* genetic backgrounds. Genetic manipulations of *Fas2* expression levels significantly change (*, one-way ANOVA, $P<0.05$) microvilli length compared to parental controls, and importantly reiterates the data obtained using the separate principal cell-specific UroGal4 driver. Solid squares indicate mean values; open circles symbolize data outliers.

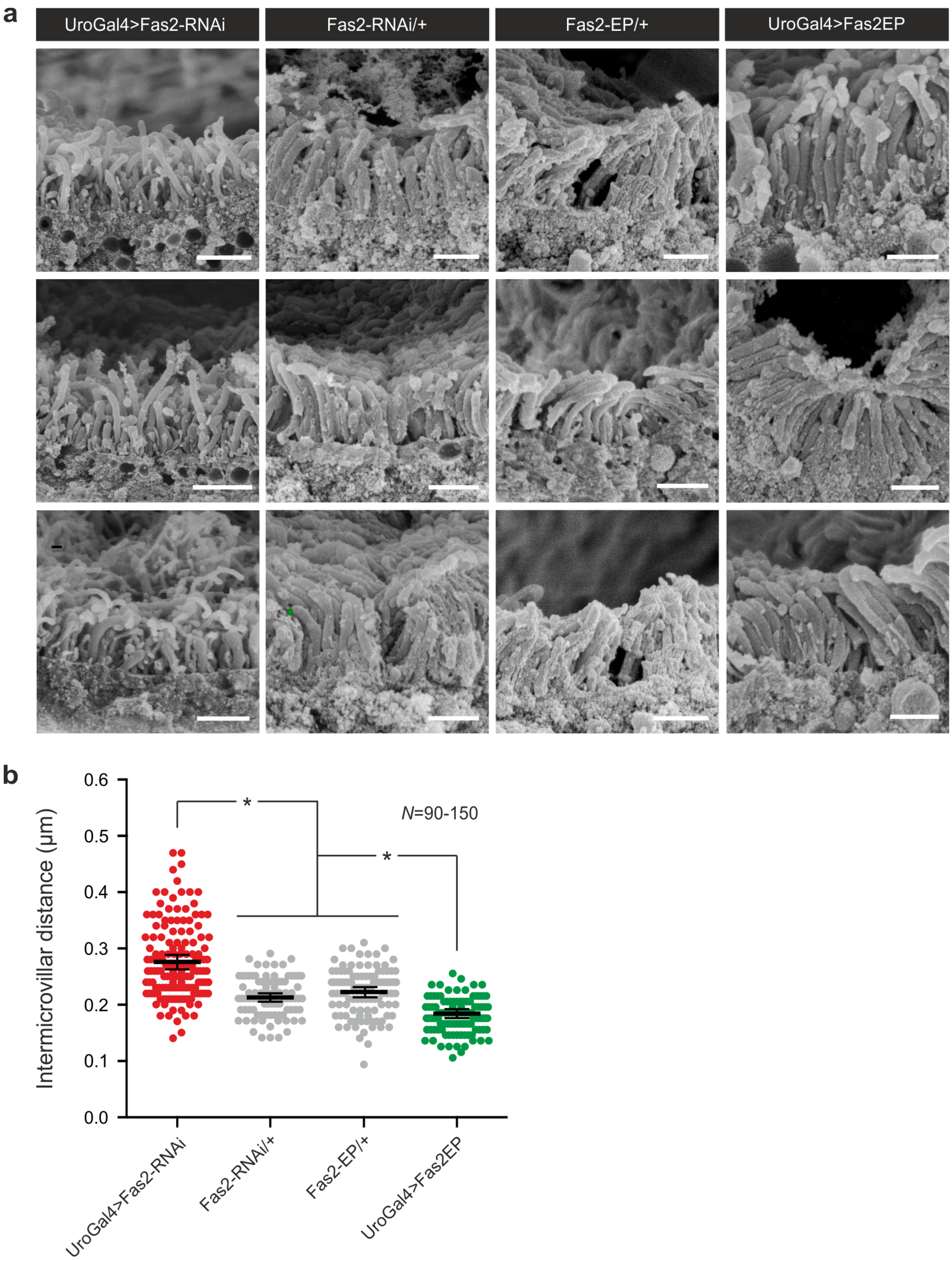

Supplementary Figure 4 | *Fas2* expression impacts brush border organisation. (a) SEM analysis of MT cross-sections (main segment) from adult *Drosophila* using the principal cell specific UroGal4 driver to drive both RNAi and overexpressor constructs. Scale bars, 1 µm. **(b)** Raw data of the quantification of intermicrovillar distance with superimposed mean ± s.e.m. in each group. *, one-way ANOVA, $P<0.05$.

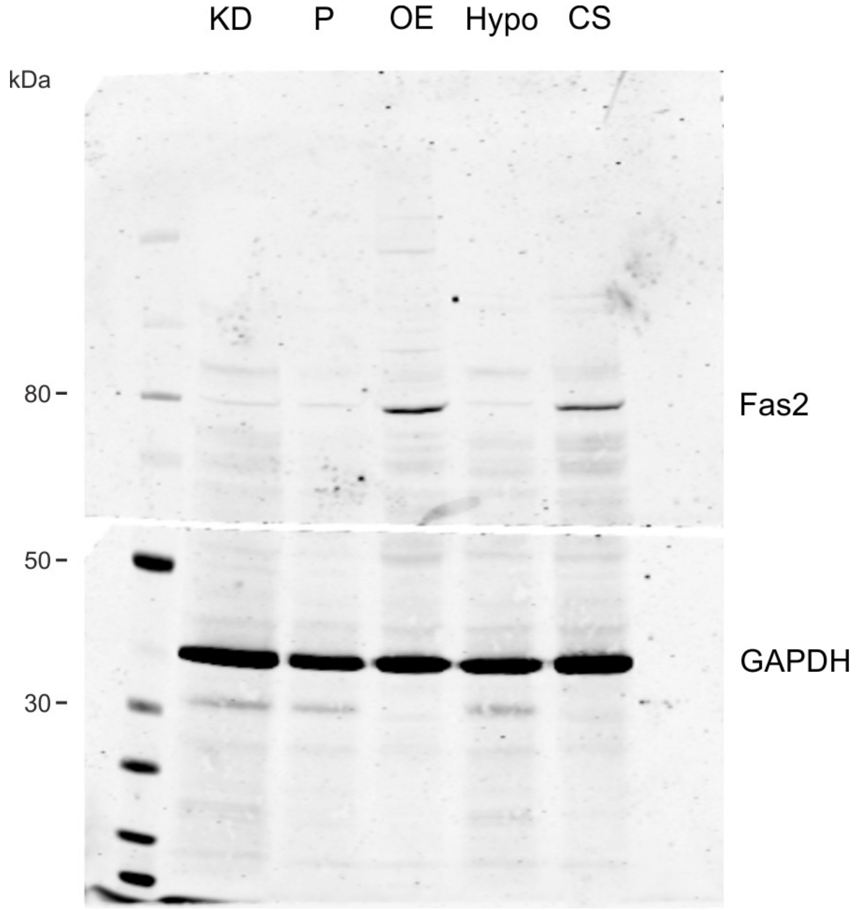

Supplementary Figure 5 | Western blot analysis of Fas2 expression. Full scan of western blot used as example in Supplementary Fig. 2b. KD, UroGal4>Fas2-RNAi; P, UroGal4; OE, UroGal4>Fas2-EP; Hypo, Fas2-EB^{112}; Canton S, CS. Fas2 protein levels were normalized against the housekeeping protein GAPDH, and values expressed as fold change relative to either parental (P) or wild type (CS).
